# Supplementary material for: Terpenes modulate bacterial and fungal growth and sorghum rhizobiome communities
Source: Microbiol Spectr. 2023 Sep 29;11(5):e01332-23. doi: 10.1128/spectrum.01332-23 (PMC10580827; doi:10.1128/spectrum.01332-23)
Supplement: Supplemental Figures — S1 to S6. [file spectrum.01332-23-s0001.docx]

**SUPPLEMENTARY MATERIALS**

**Figure S1. A)** Rhizobox with sorghum plants, shortly before harvest **B)** Rhizobox at harvest. The artificial root is visible in the middle of each box **C)** The artificial root - simulated root exudation system.

**
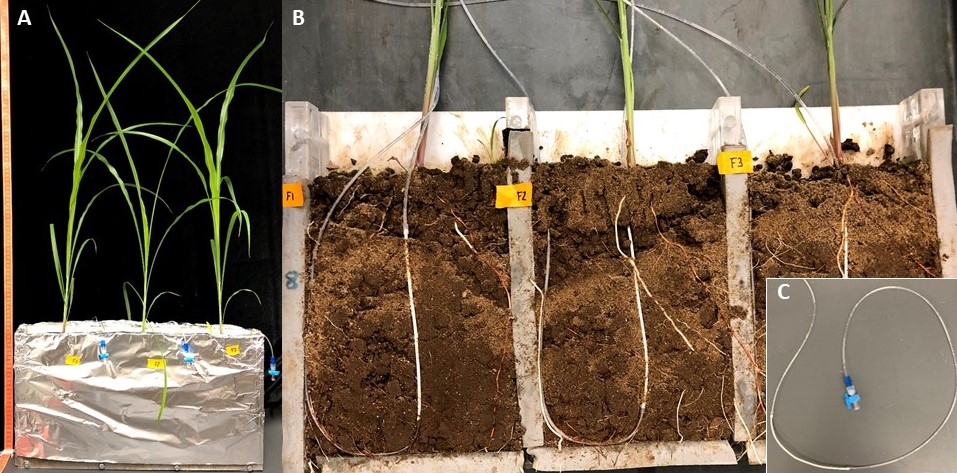
**

**Figure S2.** Relative abundance of bacterial OTUs at class level in sorghum belowground compartments treated with terpenes through artificial root. The control indicates the no-plant bare soil control.

**Figure S3.** Relative abundance of fungal OTUs at class level in sorghum belowground compartments treated with terpenes through artificial root. The control indicates the no-plant bare soil control.

**Figure S4.** Fungal isolate growth assay performed on 0.1X PDA containing 1,8-cineole, linalool, nerolidol at 100 and 200 µM, and compared with 0.1X PDA control. The bars represent colony radius normalized against the control for up to 96 hr of incubation, and the numbers above represent p-value derived from Student’s t-test comparing each treatment with the control.

**Figure S5**. Pre-screening growth curves of sorghum root bacterial isolates grown in 0.5X TSB compared with those grown in 0.5X TSB amended with either 1,8-cineole, linalool or nerolidol at 200 µM up to 25 hrs measured by cell density at OD600.

**Figure S6.** Natural log richness of sorghum artificial rhizosphere bacterial and fungal OTU after one month of terpene amendment.
