# Supplementary material for: Terpenes modulate bacterial and fungal growth and sorghum rhizobiome communities
Source: Microbiol Spectr. 2023 Sep 29;11(5):e01332-23. doi: 10.1128/spectrum.01332-23 (PMC10580827; doi:10.1128/spectrum.01332-23)
Supplement: Table S1 — Full PERMANOVA results of the terpene amendment effects. [file spectrum.01332-23-s0002.docx]

Table S1 Full PERMANOVA results of the terpene amendment effects on the microbiome of each sorghum belowground compartments

Fungal ITS

Bulk soil

Df SumsOfSqs MeanSqs F.Model R2 Pr(>F)

readNo 1 0.44205 0.44205 4.7868 0.26803 0.0004998 ***

Treatment 4 0.37611 0.09403 1.0182 0.22804 0.4282859

Residuals 9 0.83113 0.09235 0.50393

Total 14 1.64929 1.00000

---

Signif. codes: 0 ‘***’ 0.001 ‘**’ 0.01 ‘*’ 0.05 ‘.’ 0.1 ‘ ’ 1

Rhizosphere

Df SumsOfSqs MeanSqs F.Model R2 Pr(>F)

readNo 1 0.27171 0.271709 4.7141 0.21845 0.0004998 ***

Treatment 4 0.45336 0.113340 1.9664 0.36449 0.0004998 ***

Residuals 9 0.51874 0.057638 0.41706

Total 14 1.24381 1.00000

---

Signif. codes: 0 ‘***’ 0.001 ‘**’ 0.01 ‘*’ 0.05 ‘.’ 0.1 ‘ ’ 1

Aritficial rhizosphere

Df SumsOfSqs MeanSqs F.Model R2 Pr(>F)

readNo 1 0.55163 0.55163 5.8014 0.27976 0.0004998 ***

Treatment 4 0.56437 0.14109 1.4839 0.28623 0.0394803 *

Residuals 9 0.85576 0.09508 0.43401

Total 14 1.97176 1.00000

---

Signif. codes: 0 ‘***’ 0.001 ‘**’ 0.01 ‘*’ 0.05 ‘.’ 0.1 ‘ ’ 1

Root endosphere

Df SumsOfSqs MeanSqs F.Model R2 Pr(>F)

readNo 1 0.4024 0.40239 1.7743 0.11186 0.01149 *

Treatment 4 1.1539 0.28848 1.2720 0.32076 0.10395

Residuals 9 2.0411 0.22679 0.56738

Total 14 3.5974 1.00000

---

Signif. codes: 0 ‘***’ 0.001 ‘**’ 0.01 ‘*’ 0.05 ‘.’ 0.1 ‘ ’ 1

Bacterial 16S V4

Bulk soil

Df SumsOfSqs MeanSqs F.Model R2 Pr(>F)

readNo 1 0.33588 0.33588 7.7461 0.36383 0.0004998 ***

Treatment 4 0.19705 0.04926 1.1361 0.21345 0.2933533

Residuals 9 0.39025 0.04336 0.42272

Total 14 0.92318 1.00000

---

Signif. codes: 0 ‘***’ 0.001 ‘**’ 0.01 ‘*’ 0.05 ‘.’ 0.1 ‘ ’ 1

Rhizosphere

Df SumsOfSqs MeanSqs F.Model R2 Pr(>F)

readNo 1 0.59336 0.59336 11.7451 0.46425 0.0004998 ***

Treatment 4 0.23005 0.05751 1.1384 0.18000 0.3378311

Residuals 9 0.45468 0.05052 0.35575

Total 14 1.27809 1.00000

---

Signif. codes: 0 ‘***’ 0.001 ‘**’ 0.01 ‘*’ 0.05 ‘.’ 0.1 ‘ ’ 1

Artificial rhizosphere

Df SumsOfSqs MeanSqs F.Model R2 Pr(>F)

readNo 1 0.34898 0.34898 6.1533 0.25623 0.0004998 ***

Treatment 4 0.50257 0.12564 2.2154 0.36900 0.0004998 ***

Residuals 9 0.51042 0.05671 0.37477

Total 14 1.36198 1.00000

---

Signif. codes: 0 ‘***’ 0.001 ‘**’ 0.01 ‘*’ 0.05 ‘.’ 0.1 ‘ ’ 1

Root endosphere

Df SumsOfSqs MeanSqs F.Model R2 Pr(>F)

readNo 1 0.66708 0.66708 4.5084 0.25360 0.0004998 ***

Treatment 4 0.63169 0.15792 1.0673 0.24015 0.3838081

Residuals 9 1.33167 0.14796 0.50625

Total 14 2.63044 1.00000

---

Signif. codes: 0 ‘***’ 0.001 ‘**’ 0.01 ‘*’ 0.05 ‘.’ 0.1 ‘ ’ 1
